# Technical feasibility of salvage endoscopic full-thickness resection for a giant gastrointestinal stromal tumor located in low rectum after imatinib: a case report

**DOI:** 10.1093/gastro/goac078

**Published:** 2022-12-30

**Authors:** Jingyi Liu, Bing Li, Pinghong Zhou, Mingyan Cai, Yunshi Zhong

**Affiliations:** Endoscopy Center, Zhongshan Hospital of Fudan University, Shanghai, P. R. China; Endoscopy Research Institute of Fudan University, Shanghai, P. R. China; Shanghai Collaborative Innovation Center of Endoscopy, Shanghai, P. R. China; Endoscopy Center, Zhongshan Hospital of Fudan University, Shanghai, P. R. China; Endoscopy Research Institute of Fudan University, Shanghai, P. R. China; Shanghai Collaborative Innovation Center of Endoscopy, Shanghai, P. R. China; Endoscopy Center, Zhongshan Hospital of Fudan University, Shanghai, P. R. China; Endoscopy Research Institute of Fudan University, Shanghai, P. R. China; Shanghai Collaborative Innovation Center of Endoscopy, Shanghai, P. R. China; Endoscopy Center, Zhongshan Hospital of Fudan University, Shanghai, P. R. China; Shanghai Collaborative Innovation Center of Endoscopy, Shanghai, P. R. China; Endoscopy Research Institute of Fudan University, Shanghai, P. R. China; Endoscopy Center, Xuhui Hospital, Zhongshan Hospital of Fudan University, Shanghai, P. R. China

## Introduction

Gastrointestinal stromal tumors (GISTs) in adults are nearly 10%**–**30% with a potential malignant tendency [1]. Colorectal GISTs account for 5% of all cases and have a higher progression risk with a poor prognosis [2, 3]. Rectal GISTs usually need radical operations such as laparoscopy to obtain tumor-free margins. Sometimes, it is difficult for surgery to preserve the anal function because of the tumor size and location. Here, we report a case of a 70-year-old female with a giant GIST located in the lower rectum, which was removed by function-preserving endoscopic minimally invasive resection after routine imatinib therapy.

## Case report

**Figure 1.**
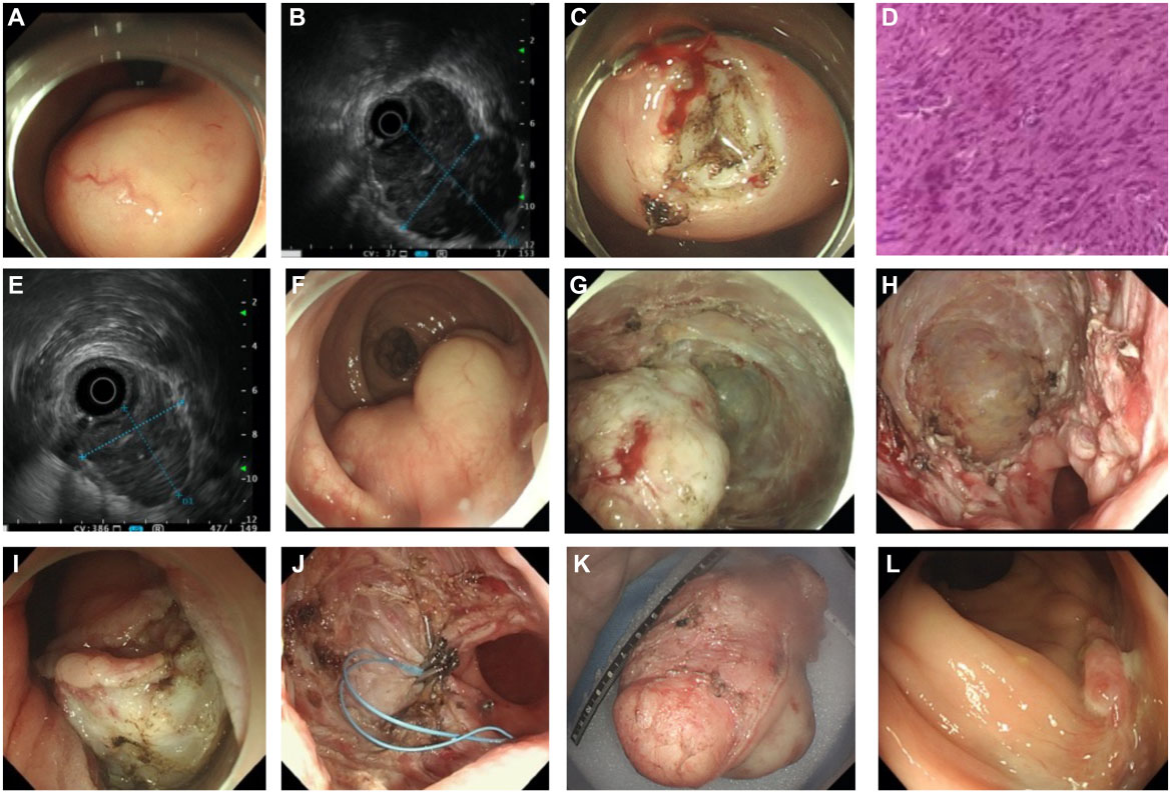
The salvage EFTR with imatinib was used to treat a giant rectal GIST in this case. (A) and (B) Colonoscopy and endoscopic ultrasound (EUS) showed a giant sub**-epithelial lesion originating from the muscularis propria; (**C) the view of the mucosal incision-assisted biopsy (MIAB); (D) representative **picture of hematoxylin and eosin staining (HE staining) for MIAB and pathology**-**confirmed GIST; (**E) and (F) EUS and colonoscopy after 10-month routine imatinib therapy; (G)–(I) the representative views of a post-resection defect of EFTR by endoscopy; (J) the appea**rance of endoscopic suturing of the defect; (**K) resected rectal giant GIST specimen en bloc; (L) representative endoscopic picture of the EFTR scar at the 3-month re-examination.

The patient was a 70-year-old female presenting with a 5-month history of rectal tenesmus and discomfort. A colonoscopy revealed a giant sub-epithelial lesion with part ulceration at 2 cm proximal to the anus, involving the rectum of ∼10 cm (Figure 1A). The surface of the GIST was easy to hemorrhage. Endoscopic ultrasound (EUS) and contrast-enhanced magnetic resonance imaging confirmed the tumor originating from the muscularis propria without any lymph node metastasis. By EUS, the maximal cross-sectional dimension of it was 7.28 × 5.71 cm (Figure 1B). Mucosal incision-assisted biopsy (MIAB) was used for tissue diagnosis (Figure 1C). The pathology of MIAB confirmed GIST and immunohistology showed that the tumor was a Ki-67 4%, CD34+, CD117+, and DOG-1+ (Figure 1D). For preserving the anal function, endoscopic full-thickness resection (EFTR) was an appropriate alternative method. However, the volume and location increased the difficulty of the operation and the risks of the EFTR of the tumor. This patient was administered 400 mg/day of imatinib in the first stage. EUS and colonoscopy were performed every 3 months for clinical assessment to resect the tumor by EFTR.

After 10-month conventional imatinib and three assessments, the volume and appearance of this tumor changed. EUS demonstrated a consistent reduction in the size of the volume during the follow-up period. Until the operation, the tumor was 5.16 × 4.32 cm in the maximal cross-sectional dimension, almost 50% of the pretreatment volume (Figure 1E). Compared with the appearance pre-imatinib, the colonoscopy observed the tumor with a soft and faint-yellow surface. The margin between the tumor and the adjacent normal rectal mucosa was apparent (Figure 1F). We planned an EFTR to resect this tumor.

The endoscopic resection was started under colonoscopy with endotracheal intubation and general anesthesia. The EFTR procedure was described as previously without intraoperative complications such as severe hemorrhaging [4]. Considering endogenous infectious complications of EFTR, prophylactic antibiotics were given during the endoscopic resection. The endoscopist observed that the blood supply of the GIST was reduced (Figure 1G and H). Eventually, en-bloc tumor resection was achieved successfully without any complications (Figure 1I–K). The post-operative pathological immunohistology showed the tumor to be a Ki-67 < 1%, SDHB+, SMA+, CD34+, CD117+, DOG-1+, and Desmin+ GIST with tumor regression grade 2 and negative margins. The wound of the EFTR recovered well 3 months post-operation under colonoscopy (Figure 1L). No recurrence had occurred at the 14-month follow-up.

## Discussion and conclusions

EFTR is an innovative endoscopic minimally invasive resection operation, being more widely used in complex colorectal lesions [5]. Compared with other endoscopic resection techniques such as endoscopic mucosal resection (EMR) and endoscopic submucosal dissection (ESD), it provides an active perforation to enable a transmural resection. EFTR is an alternative method for radical surgery in lesions considered incurable with EMR or ESD [6].

According to colorectal EFTR clinical trials, it was only used to resect colorectal sub-epithelial lesions such as neuroendocrine neoplasms rather than GISTs [4]. Due to the significantly poor prognosis for rectal GISTs, the European Society for Medical Oncology (EMSO) guideline for GISTs suggests that rectal GISTs need immediate laparoscopic/open excision on an individualized basis. In this case, the tumor was difficult to resect using common endoscopic techniques considering the volume and location. By traditional surgery, the anus function could not have been preserved for tumor-free margins.

Imatinib is used as adjuvant therapy in patients with GISTs [7]. The EMSO guideline suggests that the standard treatment for unresectable GISTs is 6- to 12-month imatinib therapy. According to a review of nine retrospective series assessing 118 patients with rectal GIST and imatinib, 5 patients (4.2%) had a complete response, 78 patients (66.1%) had a partial response, and 1 patient (0.8%) had progressive disease according to the Response Evaluation Criteria in Solid Tumors [8]. Given the factors above and the flexibility of colonoscopy, a novel strategy was performed for this patient: a salvage EFTR combined with imatinib.

Imatinib can be combined with other endoscopic therapy to treat GISTs of the digestive tract. When giant rectal GISTs grow locally toward the intestinal lumen, ESD with pre-imatinib was used to resect the GIST with anal sphincter muscle function preservation [9]. Esophageal GISTs had a significant reduction in size after the routine imatinib therapy. Esophageal GISTs after imatinib were resected using laparoscopic surgery [10]. It has been reported that a patient with esophageal GIST who underwent ESD and pre-imatinib developed exfoliative esophagitis as a complication after ESD, which may have been related to the imatinib-induced mucosal damage [10]. Endoscopic minimally invasive treatments for GISTs of the esophagus require experienced doctors.

Evaluation of the patient’s response to imatinib was performed every 3 months using EUS and colonoscopy. The volume of the tumor reduced apparently in the first and second assessments, at 76% and 56% of its pretreatment volume, respectively. However, the EUS report of the third assessment indicated a minor change compared with the second evaluation. By colonoscopy, an apparent decrease in size and a soft and faint-yellow appearance was observed. EUS with colonoscopy at certain frequencies has potential implications for confirming the proper timing to perform a salvage EFTR for a giant low-rectal GISTs after imatinib.

Compared with GISTs arising from other sites, rectal GISTs exhibit more malignant potential. In this case, comprehensive clinical and radiographic examinations were necessary to evaluate the lymph node and distant metastasis. Therefore, the imatinib-with-EFTR strategy was suggested. During pre-imatinib therapy, it is vital to perform routine assessments for evaluating tumor status. Endoscopic operations should be carried out by experienced endoscopists. Once the progression is detected, a multidisciplinary consultation is necessary for confirming the proper therapeutic strategy to improve the prognosis for patients.
